# Seroprevalence study of paratuberculosis: Johne's disease, a neglected infection in dairy herds in Apulia (southern Italy)

**DOI:** 10.1002/vro2.70020

**Published:** 2025-09-11

**Authors:** Michela Galgano, Davide Messina, Francesco D'Amico, Alessio Sposato, Elisabetta Razzuoli, Daniela Mrenoshki, Carmelinda Trisolini, Giuseppe Caldarola, Matteo Beverelli, Donato Ridolfi, Carmela Bulzacchelli, Anna Giannico, Antonio Parisi, Alessandra Tateo, Annamaria Pratelli, Domenico Buonavoglia

**Affiliations:** ^1^ Struttura complessa territoriale di Putignano (BA) Istituto Zooprofilattico Sperimentale della Puglia e della Basilicata Putignano (BA) Italy; ^2^ Servizio Veterinario di Igiene degli Alimenti di O.A. e derivati, Distretto 4, Ovest veronese Aulss 9 Scaligera Verona Italy; ^3^ Division of Veterinary Clinical Science School of Veterinary Medicine and Science Faculty of Medicine and Health Sciences University of Nottingham Loughborough UK; ^4^ S.S. Genova e Portualità Marittima Istituto Zooprofilattico Sperimentale del Piemonte, Liguria e Valle d'Aosta Genova Italy; ^5^ Department of Public Health Experimental and Forensic Medicine University of Pavia Pavia Italy; ^6^ Department of Veterinary Medicine University of Bari Valenzano Italy; ^7^ Dipartimento di Medicina di Precisione e Rigenerativa e Area Jonica (DiMePRe‐J) University of Bary Bari Italy

**Keywords:** dairy herds, infectious disease, *Mycobacterium avium* subsp. *paratuberculosis*, prevalence, southern Italy, statistical analysis

## Abstract

**Introduction:**

Paratuberculosis (PTB) is a disease caused by *Mycobacterium avium* subsp. *paratuberculosis* (MAP). In Italy, voluntary PTB control plans have been implemented; nonetheless, so far, data on the prevalence of PTB in dairy herds are fragmented and incomplete, especially in the south of the country.

**Objectives:**

The objectives were to determine the apparent (AP) and true (TP) prevalences of MAP infection, at the inter‐herd (between‐herd, BH) and intra‐herd (within‐herd, WH) levels, and the odds ratio (OR) to assess the probability of both cattle and herds testing positive in the provinces of Apulia, in southern Italy.

**Methods:**

A total of 6056 serum samples collected from 341 different dairy farms were analysed by ELISA. The number of farms and cattle required, BH and WH prevalence estimates, ORs, 95% confidence interval values (CIs) and *p*‐values (*p*) were calculated.

**Results:**

A low overall TP was estimated at both the BH level (17.14%) and WH level (0.82%). Statistically significant differences in OR (*p *< 0.05) were found for the provinces of Foggia, with a high risk of positive animals at both BH (OR = 2.6569) and WH (OR = 2.5583) levels and Taranto, with a low risk at the WH (OR = 0.5043) level.

**Conclusions:**

Our results provide the first data on the prevalence of PTB in Apulian dairy herds, according to the following criteria: (i) use of a multi‐step sample size calculation procedure considering the test's imperfection; (ii) selection of larger sample sizes of both herds; (iii) inclusion of herds and cattle from the Apulia region only recruited randomly; and (iv) analysis of the test results using non‐Bayesian approaches.

## INTRODUCTION

Paratuberculosis (PTB), also known as Johne's disease, an infectious disease caused by *Mycobacterium avium* subsp. *paratuberculosis* (MAP), was first described in 1895 in Germany.^1^ The MAP infection affects several domestic and wild mammalian species,^2^, ^3^, ^4^ including ruminants (target species) such as cattle, sheep, goats and camels and non‐ruminants (non‐target species) such as wild carnivores, pigs, rabbits, horses and birds worldwide. The MAP has been isolated from the faeces of these animals, which could therefore pose a risk to grazing livestock due to possible interaction with wild animals.^5^ However, clinical signs of the disease, which appear after long incubation periods, are evident only in ruminants (Bovidae, Cervidae) and lagomorphs, as demonstrated by the evidence of macroscopic and microscopic lesions referred to acid‐fast bacteria.^6^, ^7^, ^8^

Paratuberculosis is considered a disease of concern for animal health and trade by both the World Organisation for Animal Health (WOAH) and the European Commission. In addition, MAP raises significant public health concerns due to its possible roles in the aetiology of enteric diseases in people, such as idiopathic inflammatory bowel disease, Crohn's disease (CD) and ulcerative colitis,^9^ and in the development of Type 1 diabetes, Hashimoto's thyroiditis, multiple sclerosis and rheumatoid arthritis.^10^, ^11^ However, despite studies conducted over the past century on the link between MAP and CD, the evidence remains circumstantial and inconclusive to date.^12^

The susceptibility of cattle to MAP is closely age‐related. Calves under 1 year are the most susceptible animals in a herd, although clinical signs also appear in adult cattle (2–5 years old), manifesting as different stages of the disease.^13^, ^14^ In subclinical and clinical stages, the disease is characterised by reduced productivity, low fertility and increased susceptibility to secondary infections, such as bovine respiratory disease, mastitis and bovine viral diarrhoea,^15^ causing significant economic losses for the dairy industry.

The economic impact of PTB, however, depends on the number of infected and infectious animals,^16^, ^17^ and in Italy, the estimated economic losses associated with PTB on dairy herds exceed 200€/year/cow.^18^

In light of these observations and in particular of the economic impact on the livestock sector and the potential threat to public health, preventive health measures, such as reporting clinical cases to health authorities, should be developed and implemented in a One Health perspective to reduce bacterial load in all potential sources of MAP for animals and people. Several countries, including Italy, have implemented voluntary PTB control programmes.^19^ Epidemiological studies are based on these control programmes, defining different prevalence estimates at national and European levels. In 2013, the Italian government, at the request of the Ministry of Health, issued National Guidelines, drawn up by the National Reference Centres, for the adoption of PTB control plans and the assignment of a health ranking to dairy herds. These guidelines specify screening methods and intervention in the event of a positive test, with the aim of (i) collecting data on the occurrence of clinical cases, (ii) providing a classification of herds based on infection risk, (iii) promoting the implementation of voluntary herd control programmes, and (iv) defining a transparent procedure for the trade of cattle and their products.^20^ Currently, the national plan is voluntary and aims to support exports, reduce the spread of the infection, and create favourable conditions for informed trade of cattle and their products. Nonetheless, so far, data on the prevalence of PTB in dairy herds are fragmented and incomplete, especially in southern Italy.^21^, ^22^

The diagnosis of PTB infection is possible by performing diagnostic tests such as delayed‐type hypersensitivity (Johnin Purified Protein Derivative), interferon‐gamma release assay, polymerase chain reaction and MAP isolation.^23^ The suitability of the test depends on the clinical stage of the disease at the time of diagnosis. For the detection of antibodies in serum and milk samples, the ELISA test is the most cost‐effective serological test and is the one required by the National Guidelines for assessing the health rank of cattle herds.^24^ Although its sensitivity is generally low, it increases with the progression of the infection, and therefore, the rise in specific antibody titres during the subclinical stage of the disease.^25^, ^26^ This study aimed to estimate both the true and apparent prevalence of MAP infection at the inter‐herd (between‐herds, BH) and intra‐herd (within‐herd, WH) levels, using a commercial serological test (ELISA), to improve the limited prevalence data in the Apulia region in southern Italy. Furthermore, the probability of developing PTB (i.e., its odds ratio [OR]) has also been calculated to assess the odds of both cattle and herds testing positive in different locations. The acquired data will provide useful information for the development of prevention and control programmes to categorise and improve the health status of Apulian dairy herds.

## MATERIALS AND METHODS

### Ethical approval

The study involved a re‐use of samples already collected for health surveillance purposes. Ethical approval and informed consent from the farmers were obtained from the Ethics Committee for Animal Experimentation of the Department of Veterinary Medicine of the University of Bari (CESA‐DIMEV) under a protocol with approval no. 19/21, in compliance with the law and the ethical principle of protecting cattle as sentient creatures. The ethics committee deemed that the practices required to conduct the investigations do not cause pain, suffering, distress or prolonged harm.

### Target population

The study area is represented by the Apulia region in southern Italy. Apulia covers an area of 19,345 km^2^, including smaller islands, and comprises six provinces: Foggia in the north; Barletta–Andria–Trani and Bari in the centre; and Taranto, Brindisi and Lecce in the south.

### Test used and sample size calculations

A commercial indirect ELISA kit (ID SCREEN, Paratuberculosis Indirect ELISA kit) from IDVet (Innovative Diagnostics) was used for the detection of antibodies against MAP in the serum samples. The test also includes a confirmatory kit. The sensitivity (Se) and the specificity (Sp) of the screening and confirmatory kits were 58.2% and 99.3%, respectively.^27^

A multi‐stage sample size calculation procedure was performed, taking into account the imperfection of this test. Herds were defined as primary sampling units, while cattle within herds were defined as secondary sampling units. The method described by Humphry et al.^28^ was used to determine the number of primary and secondary sampling units to be included in the sample. To perform the calculation, using this method, the Epitools Epidemiological Calculators (Ausvet) software (Epitools—sample size to estimate a true prevalence with an imperfect test) was used. This calculated both the required primary and secondary sampling units, using the following formula twice, once for herds and once for cattle:

$$n={\left(\frac{1.960}{\mathrm{DAP}}\right)}^{2}\times \frac{\left[\left(\mathrm{SeH}\times \mathrm{BHTPE}\right)+\left(1-\mathrm{SpH}\right)\left(1-\mathrm{BHTPE}\right)\right]\left[(1-\mathrm{SeH}\times \mathrm{BHTPE}\right)-\left(1-\mathrm{SpH}\right)\left(1-\mathrm{BHTPE}\right)]}{{\left(\mathrm{SeH}+\mathrm{SpH}-1\right)}^{2}}$$

where

*n* = number to sample
1.960 = the value of the multiplier for a 95% confidence interval (CI)
DAP = desired absolute precision = the error of estimation around the prevalence that can be tolerated for the 95% confidence limits
BHTPE = the between‐herds true prevalence expected
SeH = herd sensitivity (the proportion of diseased herds that test positive)
SpH = herd specificity (the proportion of non‐diseased herds that test negative)

#### Primary sampling units calculation

The required primary sampling units (i.e., the basic elements of the sampled population, in this case primary because they are larger than the unit of interest) were calculated based on a list of dairy herds present in the National Data Base and registered in the anagrafe zootecnica (Vetinfo.it). The method previously described^28^ was used to determine the number of dairy herds to include in the sample.

On the EpiTools calculation page, the desired absolute precision was set to 0.15, while the expected effective prevalence among herds (BHTPE) was set to 50%, consistent with literature data.^21^, ^22^ Based on similar studies,^27^, ^29^ the SeH and SpH values were set at 88.9% and 42.8%, respectively, similarly detecting at least one positive cattle on the herd. Finally, a 95% CI was set among the other inputs.

Herds were considered positive if they had at least one positive animal.

The calculator estimated a minimum of 329 farms to sample, assuming an infinite population of herds.

As of 31 December 2018, there were 932 registered dairy farms in Apulia. A total of 341 farms (36.6%) were randomly sampled and distributed, as given in Table 1.

**TABLE 1:** Distribution of the primary sampling units of farms and cattle in Apulia by province.

Province	*N* farms in the province (%)	*N* farms in the province from which samples were collected (%)	*N* cattle in the province (%)	*N* tested cattle in the province^a^ (%)
Foggia	120 (12.9)	43 (35.8)	4982 (10.2)	865 (17.4)
BAT	4 (0.4)	3 (75.0)	310 (0.6)	31 (10.0)
Taranto	475 (51.0)	165 (34.7)	27,493 (56.5)	2939 (10.7)
Bari	298 (32.0)	116 (38.9)	13,520 (27.8)	1970 (14.6)
Brindisi	21 (2.3)	9 (42.9)	2122 (4.4)	168 (7.9)
Lecce	14 (1.5)	5 (35.7)	211 (0.4)	83 (39.3)
Total	932 (100)	341 (36.6)	48,638 (100)	6056 (12.5)

Abbreviation: BAT, Barletta–Andria–Trani.

^a^ All cattle tested were dairy cattle aged ≥1 year.

#### Secondary sampling units calculation

The method described above^28^ was also used to determine the number of dairy cattle to be included in the sample. The formula used for this method was the same as the one mentioned above, with SeH and SpH being substituted for the sensitivity (Se) and specificity (Sp) of the screening test, and BHTPE being substituted for the expected true prevalence within the herd (within‐herd true prevalence expected [WHTPE]).

On the EpiTools calculation page, the desired absolute precision was set to 0.15, while WHTPE was set to 3%.^21^, ^22^ Se and Sp were set to 58.2% and 99.3%, respectively, as these are the values of the screening test used, as previously mentioned.^27^ Finally, a 95% CI was set between the other inputs.

The calculator estimated a minimum sampling of 14 cattle from each farm, assuming an infinite population of cattle in a single herd.

A total of 6056 (12.5%) sera were randomly sampled from an accessible population, including sera from dairy cattle aged ≥1 year collected in 2018–2019 as part of the national brucellosis eradication programme. All farms included in the study were industrial ones, with at least 14 cattle aged ≥1 year. Because the cattle were aged ≥1 year, their age did not comply with Italian government guidelines (i.e., >3 years).^20^ As of 31 December 2018, there were 48,638 cattle aged ≥1 year in Apulia, including Frisona (50.94%), crossbred (27.82%), Bruna (12.74%) and other cattle breeds (8.5%). The area in which the sampled farms were located was mapped using QGIS software.^30^

### Laboratory analysis

Blood samples collected from the tail vein using anticoagulant‐free vacutainer tubes were stored at +4°C and delivered to the analytical laboratory of Istituto Zooprofilattico Sperimentale di Puglia e Basilicata (IZSPB), Putignano section (Bari, Italy), within 72 hours.

The samples were centrifuged (3000 × *g* for 5 minutes at 4°C), and the sera were aliquoted and frozen at 20°C until analysis.

Aliquots of each undiluted serum and the positive and negative controls provided in the kit were tested according to the test manufacturer's instructions, and the optical density (OD) readings were measured at 450 nm using an automated ELISA reader.

The OD values were transformed to Serum/Positive percentage (S/P%) according to a specific equation cited by the manufacturer: *S*/*p*% = (OD sample − ODNC)/(ODPC − ODNC).

A sample was considered positive when the *S*/*p*% value was ≥70%, doubtful if between 60% and less than 70%, and negative if *S*/*p*% ≤60%.

Serum aliquots from positive and doubtful samples were tested with the confirmation kit, and those with *S*/*p*% ≥70% values were considered positive.

### Prevalence calculation

To calculate the apparent prevalence between‐ and within‐herds and to estimate the 95% CIs, the Epitools Epidemiological Calculators (Ausvet) software (Epitools—Estimated true prevalence and predictive values from survey testing) was used. This software calculates the apparent prevalence, using the formula (*a* + *b*)/(*a* + *b* + *c* + *d*), and the relative Agresti–Coull^31^ confidence limits based on the method described by Brown et al.^32^ In the apparent prevalence formula, *a* represents true‐positive farms or cattle, *b* false‐positive farms or cattle, *c* false‐negative farms or cattle and *d* true‐negative farms or cattle.

The estimate of the true prevalence between‐herds (BW) and within‐herd (WH) was calculated using the method described by Rogan and Gladen,^33^, and confidence limits for this estimate were calculated using the method described by Reiczigel et al.^34^

#### Between‐herd prevalence calculation

On the EpiTools relevant page to the calculation, the sample size was set to 341. Se and Sp were set to 58.2% and 99.3%, respectively, as these are the values of the screening test used. Finally, a 95% CI was set among the other inputs. The Agresti–Coull^31^ method was set as the CI as recommended by Thrusfield,^35^ as *n* > 40.

#### Within‐herd prevalence calculation

On the EpiTools relevant page to the calculation, the sample size was set to 6056, as this is the number of dairy cattle tested. Se and Sp were set to 58.2% and 99.3%, respectively, as these are the values of the screening test used. Finally, a 95% CI was set among the other inputs. Blaker^36^ method was set as a CI as recommended by Reiczigel et al.^34^

### Odd ratios calculation according to the location of positives

Two‐by‐two contingency tables were constructed to conduct statistical analysis of positive herds and cattle, and their location. The statistical analysis consisted of OR calculations and their related 95% CIs and *p*‐values (*p*). Odds ratios are measures of association between exposures and outcomes. They represent the ratio of the odds of outcomes occurring given specific exposures and the odds of outcomes occurring in the absence of those exposures.^37^ We used OR to estimate the odds of both herds and cattle being positive (outcomes) given different locations (exposures). We considered that, for an OR = 1, the location did not affect the odds of herds or cattle being positive; for an OR > 1, location increased the odds of herds or cattle being positive; for an OR < 1, the location reduced the odds of herds or cattle being positive.^37^ The OR calculation was performed in two steps. In the first step, Microsoft Excel for Microsoft 365 MSO (Version 2412 Build 16.0.18324.20092) 64‐bit was used, and positive herds and cattle were classified according to the so‐constructed two‐by‐two contingency tables to assess the association between the relevant outcome and their exposure. The following example shows how the two‐by‐two tables were constructed (Table 2).

**TABLE 2:** Example of a two‐by‐two table used to classify cattle according to test results (outcome) and location (exposure).

Location concerned (exposure), e.g., Bari			
Test result (outcome)	Bari	Non‐Bari	Total
Positive	23	48	71
Negative	1947	4038	5985
Total	197	4086	6056

In the second step, the derived data were entered in MedCalc Software Odds ratio calculator version 23.0.5.^38^ The OR, 95% CIs and *p‐*values were calculated to assess the association between positive herds and cattle and geographic location. As a result of the example shown in Table 2, dairy cattle in the province of Bari had an OR equal to 0.9938 (95% CI: 0.6028–1.6384; *p* = 0.9805), meaning that they were 0.9938 times more likely to develop Johne's disease than those in other provinces in Apulia (i.e., non‐Bari).

## RESULTS

Based on the size of the Apulian livestock, a sample of 6056 (12.5%) dairy cattle in 341 (36.6%) herds was identified for testing. Among these, 36 herds reported at least one positive dairy cattle and a total of 71 cattle tested seropositive for MAP as confirmed by the ELISA confirmatory kit used. At the manufacturer's suggested cut‐off, an apparent BH prevalence of 10.56% (95% CI: 7.69–14.30) and an apparent WH prevalence of 1.17% (95% CI: 0.93–1.48) were identified. However, adjusting the apparent prevalence for the relative sensitivity and specificity of the ELISA (sensitivity = 58.2%, specificity = 99.3%), using the standard method, the estimate of the true BH prevalence estimate was 17.14% (95% CI: 12.22–23.60), while the estimated true WH prevalence estimate was 0.82% (95% CI: 0.40–1.85). All results are summarised in Table 3.

**TABLE 3:** Apparent and true prevalence estimates of MAP infection between‐herds and within‐herds in the provinces of Apulia.

	Between‐herd AP and TP estimates		Within‐herd AP and TP estimates	
Province	AP (Agresti–Coull 95% CI %)	TP (Blaker 95% CI %)	AP (Agresti–Coull 95% CI %)	TP (Blaker 95% CI %)
Foggia	20.9 (11.2–35.4)	35.2 (18.7–60.0)	2.4 (1.6–3.7)	3.0 (1.6–5.2)
BAT	0 (−5.6 to 61.8)	−1.2 (−1.2 to 96.4)	0 (−2.1 to 13.1)	−1.2 (−1.2 to 18.0)
Taranto	7.9 (4.6–13.1)	12.5 (6.9–21.4)	0.8 (0.5–1.2)	0.1 (−0.3 to 0.8)
Bari	9.5 (5.2–16.3)	15.3 (8.1–26.9)	1.2 (0.8–1.8)	0.8 (0.1–1.8)
Brindisi	22.2 (5.3–55.7)	37.4 (9.8–94.0)	1.2 (0.1–4.5)	0.9 (−0.7 to 6.2)
Lecce	20.0 (2.0–64.0)	33.6 (0.6–107.4)	2.4 (0.2–8.9)	3.0 (−0.1 to 13.3)
Total	10.6 (7.7–14.3)	17.1 (12.2–23.6)	1.2 (0.9–1.5)	0.8 (0.4–1.4)

*Note*: All values less than 0 should be considered 0. Statistically significant odds ratios (*p *< 0.05) were observed only for the provinces of Foggia and Taranto (Table 4).

Abbreviations: AP, apparent prevalence; BAT, Barletta–Andria–Trani; CI, confidence interval; MAP, *Mycobacterium avium* subsp. *paratuberculosis*; TP, true prevalence.

Statistically significant odds ratios (*p *< 0.05) were observed only for the provinces of Foggia and Taranto (Table 4).

**TABLE 4:** PTB‐positive herds and cattle's ORs according to locations.

Provinces	Positive herds’ ORs according to locations				Positive cattle's ORs according to locations			
	*N* (%)	OR	95% CI (min–max)	*p*‐value	*N* (%)	OR	95% CI (min–max)	*p*‐value
Foggia	9/43 (20.9)	2.7^*^	1.2–6.1^*^	0.0217^*^	21/865 (2.4)	2.6^*^	1.5–4.3^*^	0.0003^*^
BAT	0/3 (0)	1.2	0.1–23.4	0.9117	0/31 (0.0)	1.3	0.1–21.8	0.8453
Bari	11/116 (9.5)	0.8	0.4–1.8	0.6433	23/1970 (1.2)	1.0	0.6–1.6	0.9805
Taranto	13/165 (7.9)	0.6	0.3–1.2	0.1227	23/2939 (0.8)	0.5^*^	0.3–0.8^*^	0.0072^*^
Brindisi	2/9 (22.2)	2.5	0.5–12.5	0.2641	2/168 (1.2)	1.0	0.2–4.2	0.9824
Lecce	1/5 (20.0)	2.2	0.2–19.8	0.4990	2/83 (2.4)	2.1	0.5–8.8	0.3029

Abbreviations: BAT, Barletta–Andria–Trani; CI, confidence interval; OR, odds ratio; PTB, paratuberculosis.

* Significant result.

Herds in the province of Foggia had a 2.6569‐fold increased OR of developing MAP infection (95% CI: 1.1534–6.1201; *p* = 0.0217) compared to those in the other provinces of Apulia. This was the highest and the only statistically significant OR (*p *< 0.05), followed by the provinces of Brindisi, Lecce, Barletta–Andria–Trani (BAT), Bari and Taranto for which the data were not considered statistically significant (*p *> 0.05).

Similarly, cattle from the province of Foggia had a 2.5583‐fold increased OR of developing MAP infection (95% CI: 1.5288–4.2810; *p* = 0.0003) times the odds compared to those from other provinces in Apulia. This was the highest and one of two statistically significant ORs (*p *< 0.05), followed by the provinces of Lecce, BAT, Brindisi and Bari, although without statistical significance (*p *> 0.05) for these. The province of Taranto, whose cattle were 0.5043 times more likely to develop MAP infection (95% CI: 0.3060–0.8312; *p* = 0.0072) compared to those in the other province of Apulia, had the lowest and the other of the two statistically significant (*p *< 0.05) OR. Cattle from the province of Brindisi, which had a 1.0161 (95% CI: 0.2470–4.1797; *p* = 0.9824) times higher odds of developing MAP infection than those from other provinces in Apulia, fell in between the highest and lowest ORs in Foggia and Taranto, respectively.

Interestingly, from this OR analysis, both herds and cattle from the province of Foggia were more likely to be positive, as evidenced by the relevant OR > 1 and *p *< 0.05, suggesting a possible increased risk of MAP infection in this province. Whereas cattle from the province of Taranto were less likely to be positive, as evidenced by the relevant OR < 1 and *p *< 0.05, suggesting a possible reduced risk of infection.

## DISCUSSION

Paratuberculosis can cause significant economic losses in the cattle industry and potential public health problems.^10^, ^11^, ^17^, ^39^, ^40^, ^41^ Although the zoonotic role of MAP has not yet been established, the awareness that its presence in the food chain could have serious implications for both public health and international trade makes its control even more urgent, should MAP be definitively identified as a zoonotic agent. In light of the not entirely unlikely hypothesis that MAP may be definitively identified as a zoonotic agent, we assessed the prevalence of MAP infection in Apulia, a region of southern Italy that is still under‐researched. The infection level was estimated at both the WH and BH levels to improve prevalence data, thus providing epidemiological data useful to the scientific community and the region for designing regional prevention and control programmes aimed at eliminating MAP from local farms. To properly conduct the investigation, the sample size for screening was first estimated, ensuring it was as representative as possible of the Apulia region based on the resources available and the characteristics of the test used. This resulted in the involvement of 341 different dairy cattle farms (36.6%) and 6056 dairy cattle (12.5%). Furthermore, the probability of developing MAP infection (OR) was also calculated to assess the odds of both cattle and herds testing positive in different locations. The OR is indeed an important parameter in disease control programmes for the identification of a subset of herds with a high probability of being free from infection. Animals free from MAP infection would, in fact, represent a source of low‐risk livestock and dairy products marketable in Europe and beyond, protecting both animals (low risk of disease spread) and people (low risk for the consumer) in a One Health perspective.

In our study, less than a quarter (17.14%) of the tested herds were seropositive for PTB. These values are consistent with the estimated prevalence range at national (19.9%–52.8%)^21^, ^42^, ^43^ and international levels, such as in the UK (17%).^44^ In particular, the BH seroprevalence of MAP found in Apulia is comparable to that observed in another study conducted in southern Italy (19.9%)^21^ and Ireland (28%),^45^ while it is significantly lower than seroprevalence reports from northern (43.7%)^42^ and southern (46%)^22^ Italy and across Europe (>50%),^44^ Canada (46.4%),^46^ and higher than that reported in Norway (10%).^44^ In contrast, WH seroprevalence (0.82%) is higher than that recorded in Norway (0.1%),^44^ and lower than that recorded in prevalence estimate reports conducted in northern (2.6%)^42^ and southern (3%)^22^ Italy, and that of the UK (2.46%–5.6%),^47^, ^48^ Ireland (3.2%)^45^ and the United States (14%).^49^

As the accuracy and precision of prevalence estimates can be significantly influenced by sampling methods, the diagnostic tests used and the variability of the condition within the population under examination,^24^ these differences could be attributed to various causes. It is known that the diagnostic sensitivity of a test varies depending on the stage of the disease: the characteristic course of PTB, with relatively very low anti‐MAP antibody titres already after a few months from infection, makes the identification of infected cattle extremely difficult. Furthermore, estimating the Se and Sp from diagnostic tests for MAP infection is a complex issue, also due to the absence of a reference standard for determining the infection status of cattle and herds. It is therefore complex to compare prevalence estimates derived from different population series, especially when different sampling methods and diagnostic tests are used.^50^ However, to limit data bias in this study, the true and apparent prevalences of infection were estimated for the first time in the region of Apulia according to the following criteria: (i) use of a multi‐step sample size calculation procedure considering the imperfection of the test^28^; (ii) selection of larger sample sizes of both herds (36.6% of the herds registered in Apulia on 31 December 2018) and cattle (12.5% of the dairy cattle aged over 1 year registered in Apulia on 31 December 2018); (iii) inclusion of herds and cattle from the Apulia region only recruited randomly; and (iv) analysis of the test results using non‐Bayesian approaches.^31^, ^32^, ^33^, ^34^, ^35^

The seroprevalence values recorded in this study can be regarded as sufficiently representative of the spread of MAP within the Apulia region and its various provinces, and represent the only truly meaningful account of the territorial epidemiological situation. In fact, considering that the studies on the prevalence of MAP infection in Apulian dairy cattle in the literature are limited to a few previous studies,^21^, ^22^ the present study enriches the existing epidemiological databases on Italian territory available to the scientific community. In addition, this information highlights the importance of implementing appropriate prevention and risk management strategies to mitigate potential transmission through the consumption of dairy products derived from infected animals. To this end, OR analysis allowed for a preliminary risk assessment and highlighted the provinces of Foggia and Taranto as those with the highest and lowest risk of MAP infection, respectively, making these data of tangible value for infection control in these provinces. Therefore, it is worth emphasising the validity of such analysis used in this preliminary study in providing valuable support for the design of a targeted regional surveillance and prevention plan. This way, livestock can also be qualified and dairy products certified, thus reassuring consumers about the food safety of the finished product.

However, this is certainly a preliminary study that must be interpreted in the context of its limitations, such as: (i) the lack of information on herd management that could have helped to identify risk factors associated with MAP infection and the calculation of OR; (ii) the desired absolute precision of the multi‐stage sample size calculation^28^ set to 0.15, meaning that the error of estimation around the prevalence tolerated for the 95% confidence limits is ±15% and, therefore, the width of the 95% CI is 30%^35^; (iii) the age of the cattle that did not comply with Italian government guidelines (i.e., >3 years),^20^ as it included cattle ≥1 year; and (iv) the absence of anamnestic data and age‐specific information that could have been incorporated into the study variables, reflecting the characteristics of the test based on the stage of infection. Points (iii) and (iv) are related to privacy restrictions on the use of sera from official prophylaxis programmes, and for this reason, the results for some provinces did not reach statistical significance.

Nevertheless, further studies are needed to assess the OR based on transmission risk factors, to strengthen the set of data for the provinces of Foggia and Taranto and to assess the risk of infection in the remaining Apulian provinces. This could allow for the development of an effective control and eradication plan, clearly requiring any further research to also consider herd characteristics, such as average herd size, access to pasture, and a mix of industrial and family farm management. The plan should also take into account potential contact with wildlife and the use of shared grazing areas.

In conclusion, this study provides a useful preliminary assessment of the prevalences of MAP infection in the provinces of Apulia in southern Italy, where a low overall TP was estimated at both BH level (17.14%) and WH level (0.82%), and statistically significant differences in OR (*p *< 0.05) were found for the provinces of Foggia, with a high risk of positive animals at both BH (OR = 2.6569) and WH (OR = 2.5583) levels and Taranto, with a low risk at WH (OR = 0.5043) level. However, further studies are needed at the national level—including data from different regions—as well as at the international level, to obtain a more accurate estimate of the disease prevalence in each country and to provide useful data for the global economic and public health management of the problem.

## AUTHOR CONTRIBUTIONS

**Michela Galgano**: contributed to conceptualisation, data curation, investigation, methodology, project administration, validation, visualisation, writing—original draft, writing—review and editing. **Davide Messina**: contributed to conceptualisation, data curation, formal analysis, methodology, project administration, software, validation, visualisation, writing—original draft, writing—review and editing. **Francesco D'Amico**: contributed to project administration, visualisation, writing—review and editing. **Alessio Sposato**: contributed to data curation, investigation, visualisation. **Elisabetta Razzuoli**: contributed to visualisation, writing—review and editing. **Daniela Mrenoshki**: contributed to visualisation. **Carmelinda Trisolini**: contributed to data curation and software. **Giuseppe Caldarola**: contributed to data curation and software. **Matteo Beverelli**: contributed to resources. **Donato Ridolfi**: contributed to investigation and methodology. **Carmela Bulzacchelli**: contributed to investigation and methodology. **Anna Giannico**: contributed to investigation and methodology. **Antonio Parisi**: contributed to project administration, resources, supervision, validation. **Alessandra Tateo**: contributed to visualisation. **Annamaria Pratelli**: contributed to project administration, resources, supervision, validation, writing—review and editing. **Domenico Buonavoglia**: contributed to conceptualisation, investigation, project administration, resources, supervision, validation, writing—review and editing.

## CONFLICTS OF INTEREST

The authors declare they have no conflicts of interest.

## ETHICS STATEMENT

The study involved a re‐use of samples already collected for health surveillance purposes. Ethical approval and informed consent from the farmers were obtained from the Ethics Committee for Animal Experimentation of the Department of Veterinary Medicine of the University of Bari (CESA‐DIMEV) under a protocol with approval no. 19/21, in compliance with the law and the ethical principle of protecting cattle as sentient creatures. The ethics committee deemed that the practices required to conduct the investigations do not cause pain, suffering, distress or prolonged harm.

## Data Availability

The data that support the findings of this study are available from the corresponding authors upon reasonable request.
